# Bacterial contamination of the hands of food handlers as indicator of hand washing efficacy in some convenient food industries in South Africa

**Published:** 2014

**Authors:** Lambrechts AA, Human IS, Doughari JH, Lues JFR

**Affiliations:** 1 Lambrechts AA, Faculty of Applied Sciences, Department of Environmental Health, Cape Peninsula University of Technology, Cape Town, South Africa.; 2 Human IS, Faculty of Applied Sciences, Department of Environmental Health, Cape Peninsula University of Technology, Cape Town, South Africa.; 3 Doughari JH, Faculty of Applied Sciences, Department of Environmental Health, Cape Peninsula University of Technology, Cape Town, South Africa.; 4 Lues JFR, Department of Life Sciences, Central University of Technology, Bloemfontein, South Africa.

**Keywords:** Hand washing, Hygiene, Food handlers, Contamination, *S. aureus*, *E. coli*

## Abstract

***Background and Objectives:*** Hands of ready-to-eat food service employees have been shown to be vectors in the spread of foodborne disease, mainly because of poor personal hygiene and accounting for approximately 97% of food borne illnesses in food service establishments and homes. Our objective was to evaluate the efficacy of hand washing practices and sanitation before commencing work among food handlers in the convenient food industry in Gauteng, South Africa.

***Methods:*** A total of 230 samples were collected, involving 100% of the food handlers, in 8 selected convenient food outlets with their main focus on preparing ready-to-eat foods. The workers’ cleaned and disinfected dominant hands were sampled for Total Plate Count (TPC), *Staphylococcus aureus* and *Escherichia coli*. Bacteria were isolated and counted using standard methods.

***Results:*** The highest bacterial count from the hand samples was 7.4 x 10^3 ^cfu.cm^-2 ^and the lowest showed no detectable growth. Although hands with a count of 0 cfu.cm^-2 ^were found in all of the plants, the results indicated that all the plants exceeded the legal limit for food surfaces or hands of < 100 cfu.cm^-2^ when the average bacterial counts on hands were compared. Sixty percent of the TPC analysed exceeded the legal limit and only 18% of the food handlers had no bacteria detectable on their hands. One sample tested positive for *E. coli* and *S. aureus* could not be detected on the hands of any of the food handlers.

***Conclusion:*** The study revealed that hand hygiene is unsatisfactory and may have serious implications for public health due to contamination of food from food handlers’ hands. This therefore underlined the importance of further training to improve food handlers’ knowledge of good hand washing practices.

## INTRODUCTION

The hands of ready-to-eat food service employees have been shown to be vectors in the spread of foodborne disease, mainly because of poor personal hygiene. Howes *et al.*^1^ state that improper food handler practices contributed to approximately 97% of foodborne illnesses in food service establishments and homes. Statistical evidence indicates that food poisoning caused by the catering industry is 70% higher than that caused by any other sector.^2^

Hand washing is a fundamental precautionary measure to protect against the spread of disease and is one of the primary practices to reduce the transfer of bacteria, whether from person to person, or from person to food contact surfaces.^3^ The main reason for limiting contact between ready-to-eat foods and people’s hands is to prevent the transfer of viruses and bacteria that are already present in human bodies.^4^ Furthermore, it was established that a food worker’s unwashed hands can transmit pathogens, especially faecal pathogens, to food products after a visit to the toilet. Investigations of foodborne illness outbreaks have shown that poor personal hygiene, primarily ineffective hand washing, is an important contributor to foodborne illness, second only to inadequate temperature controls of food.^5^ According to Government Regulation 962 of 2012, promulgated under the Foodstuffs, Cosmetics and Disinfectants Act, No. 54 of 1972 of Republic of South Africa^6^, it is a requirement for food handlers to wash their hands with soap and hot and/or cold water before handling any food product or container or working in a food facility. This regulation further stipulates that a maximum of 100 viable organisms are allowed per cm^2 ^after cleaning and sanitation of food contact surfaces has occurred. For the purpose of this study, the same standard **was** applied to workers’ hands, as they come into direct contact with the ready-to eat food produced. Annexure B of the *Guidelines for Environmental Health Officers on the Interpretation of Microbiological Analysis Data of Food*^7^ for South Africa does not make provision for maximum counts related to *E. coli *and* S. aureus* on food contact surfaces or hands, but the organisms must be absent in all food products.

This study was done to evaluate the efficacy of hand washing practices and sanitation amongst food handlers before they commence working in convenience food plants in the Gauteng Province of South Africa. The study should add to the existing body of knowledge on hand washing and sanitation in the ready-to-eat food industry.

## METHODS

***Sampling protocol: ***A 20% sample was randomly selected from 40 convenient food outlets in Gauteng, which were selected because their predominant focus is on preparing ready-to-eat foods.^8^ The samples were collected from workers’ cleaned and disinfected dominant hands (after their normal washing and disinfection), which are normally in direct contact with the food using the swab SABS method 762 after each worker had passed through the hand washing area before commencement of work. A total of 230 microbiological samples were collected from 100% of the food handlers at the 8 convenient food plants (Table-I). Samples were transported to the laboratory on ice and then immediately subjected to microbiological analysis to determine the Total Plate Count (TPC), and to determine the presence and prevalence of *Staphylococcus aureus* and *Escherichia coli. *In order to ensure consistency of workers’ normal practices in washing and disinfection, they had no prior knowledge of the planned sampling runs. Furthermore, the samples were collected on working days and adequate time was allowed for workers to clean and sanitize their hands. Results are the means of duplicate analyses.

***Microbiological analysis***

***Total Plate Count (TPC): ***For TPC determination, the conventional pour plate technique as described by ISO Method 4833^10^ was used with slight modification. Briefly, swab samples collected were first inoculated into 5 ml of nutrient broth (NB) in test tubes followed by serial dilution of each tube, after which 0.1 ml of the 10^-3^ dilution was transferred into sterile Petri dishes. To each of this broth culture dilution, sterile nutrient agar (NA) was dispensed; the plates were properly mixed, allowed to solidify and then incubated at 30°C for 72 h. After incubation, the TPC was determined and was expressed as colony forming units (cfu/ml).

***Recovery and enumeration of E. coli and S. aureus: ***For recovery and enumeration of *E. coli *and* S. aureus*, the spread plate technique of ISO 16649-2^11^ was used. Swab samples collected were soaked in 5 ml of NB as for TPC and serially diluted. From this dilution 0.1 ml of the up to 10^-3 ^dilution were spread inoculated onto Petri dishes containing solidified sterile MacConkey agar (MCA) for *E. coli *and Baird-Parker agar medium for *S. aureus. *The culture plates were then incubated at 37°C for 72 h. After incubation all the bacterial colonies were enumerated using the colony counter and further identification of *E. coli *and *S. aureus *was carried out on the basis of morphology, cultural characteristics and biochemical tests.^12^ Identification of *S. aureus *and* E. coli *was further carried out using the latex agglutination test kits StaphTEX™ Blue Latex, (Hardy diagnostics, USA) and *E. coli* PRO™ Latex (Hardy diagnostics, USA) respectively.

***Data analysis: ***Results were analysed in collaboration with the Cape Peninsula University of Technology’s Corrie Uys, Statistician, Centre for Postgraduate Studies, and results were expressed as frequencies and percentages in Tables and Graphs. 

## RESULTS

**Fig.1:**
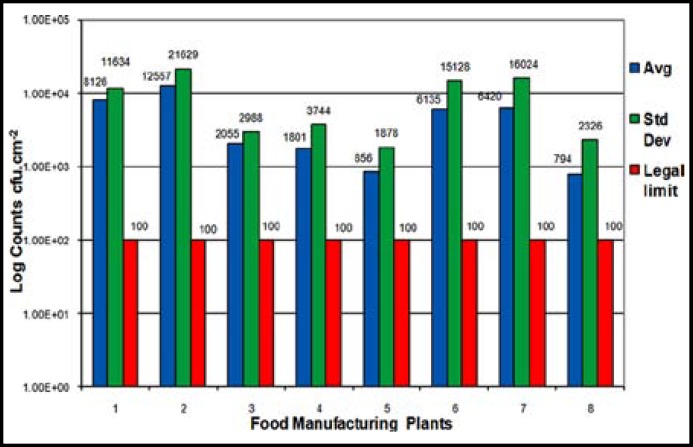
Comparison between the standard deviation and the average Total Plate Count versus the legal limit of < 100 cfu.cm^-2^.

According to Fig.1, the highest bacterial count (TPC) found from the hand samples was 7.4 x 10^3^ cfu.cm^-2^ (Plant 2) and the lowest had no detectable growth. Although hands with a count of 0 cfu.cm^-2 ^were found in all the plants, the results indicated that all the premises sampled exceeded the legal limit of < 100 cfu.cm^-2^ when the average bacterial counts on hands were compared. The normal data distribution, standard deviation and average bacterial count considerably exceeded the legal limit.^13^ Except at plants 5 and 8, the average bacterial count was higher than 10^3^ cfu.cm^-2^ and one premises (Plant 2) exceeded 10^4^ cfu.cm^-2^. 

## DISCUSSION

**Fig.2:**
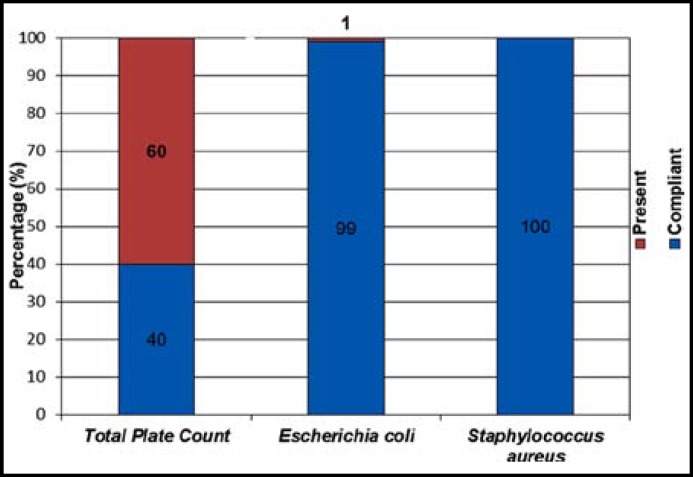
Percentage compliance of Total Plate Count (< 100 cfu.cm^-2^), *Escherichia coli* and *Staphylococcus aureus* samples collected from workers’ cleaned and sanitised dominant hand surfaces in 8 convenient food manufacturing plants

 The primary action of hand washing is the mechanical removal of viable transient microorganisms, whereas the primary action of antimicrobial soap includes both mechanical removal and killing or inhibition of both transient and resident flora.^14^ This is an indication of insufficient hand washing and sanitation, as one would expect a significantly reduced bacterial count on the workers’ hands after they have cleaned and sanitised them. Paulson^15^ & Raspor^16^ reported the importance of management training of all employees in the use of effective hand washing procedures, and that the safety of food chain supply can easily be broken proper enforcement these procedures. Sixty percent of the TPC samples analysed exceeded the legal limit (< 100 cfu.cm^-2^) stipulated by the Foodstuffs, Cosmetics and Disinfectants Act for food contact surfaces^6^ (Fig.2).

**Table-I:** Distribution of samples collected from hands

***Convenience Food Manufacturing Plants***	1 ***Total PlateCount***	2 ***Escherichia coli***	3 ***Staphylcoccus aureus***	***Total no of samples per plant***
1	9	9	9	27
2	12	11	10	33
3	11	8	8	27
4	13	12	11	36
5	9	7	6	22
6	14	12	9	35
7	10	9	5	24
8	10	9	7	26
Total	**88**	**77**	**65**	**230**

1 ISO method 4833 (International Organisation for Standardization, 2003)

2 ISO method 16649-2 (International Organisation for Standardization, 2001)

3 ISO method 6888-1 (International Organisation for Standardization, 1999)

Only one sample (1%: Plant 6) of the hand samples analysed, tested positive for* E. coli *(Fig.2). As* E. coli* is found in the intestinal tract of both humans and animals, finding this organism in ready-to-eat foods is generally viewed as an indication of faecal contamination. Faecal contamination, in turn, indicates that other harmful organisms, whether they be bacterial genera (*Salmonella, Shigella, Campylobacter*), viral (Hepatitis A, norovirus, rotavirus) or helminthic or protozoal parasites (*Taenia, Toxoplasma, Cryptosporidium, Giardia*), could be present.^17^ In addition, the test for generic *E. coli *may also point to highly pathogenic strains of *E. coli* that have the ability to cause diarrhoea as well as systemic disease, resulting in multi-organ failure and death (*E. coli* 0157:H7).^18^ It is for these reasons that the confirmation of *E. coli* in ready-to-eat food is followed by an automatic recommendation for a thorough review of the constituent ingredients, as well as finished product re-testing and task-oriented training of those individuals involved in the preparation of those specific ready-to-eat food items.

Throughout the eight food premises, *S. aureus* could not be detected on the hands of food handlers (Fig.2). *S. aureus *and coagulase-negative *Staphylococci* (CNS) inhabit the human skin and mucous membranes, where they exist mostly as commensal flora.^19^ Humans are the natural carriers of *S. aureus* and the organism can be found in a healthy human population.^20^ The onset of symptoms in staphylococcal food poisoning is usually rapid and in many cases acute, depending on the individual’s susceptibility to the toxin, the amount of contaminated food eaten, the amount of toxin in the food ingested and the general health of the victim. *Staphylococci* exist in air, dust, sewage, water, milk and food or on food equipment, environmental surfaces, humans and animals. Humans and animals are the primary reservoirs of *Staphylococci*.^20^

## CONCLUSION

Results of this study revealed the unsatisfactory level of the hand hygiene among the food handlers investigated and underlines the need to improve food handlers’ hygiene knowledge by focusing on hand washing practices. It is of utmost importance that high standards of sanitation, cleanliness and good housekeeping be maintained at all times and any laxness in this regard may result in a serious epidemic or infection.^21^ Employees should be trained on how to handle food as well as on sanitation and hand washing techniques, as bacteria from cuts, infections, boils or other communicable diseases may cause food poisoning.^22^ People involved with every stage of food production, from farm to fork must take responsibility to prevent infections and destroy pathogens.^29^

## Authors contribution:

**Lambrechts AA: **Experimentation, data collection and manuscript writing.

**Human IS: **Designing, co-writing and supervision of the project.

**Doughari JH: **Critically reviewed the manuscript for final publication.

**Lues JFR: **Co-supervision.
